# Pharmacologic Blockade of JAK1/JAK2 Reduces GvHD and Preserves the Graft-Versus-Leukemia Effect

**DOI:** 10.1371/journal.pone.0109799

**Published:** 2014-10-07

**Authors:** Jaebok Choi, Matthew L. Cooper, Bader Alahmari, Julie Ritchey, Lynne Collins, Matthew Holt, John F. DiPersio

**Affiliations:** 1 Division of Oncology, Department of Medicine, Washington University School of Medicine, St. Louis, Missouri, United States of America; 2 BRIGHT Institute, and Molecular Imaging Center, Mallinckrodt Institute of Radiology, and Department of Cell Biology & Physiology, Washington University School of Medicine, St. Louis, Missouri, United States of America; Beth Israel Deaconess Medical Center, Harvard Medical School, United States of America

## Abstract

We have recently reported that interferon gamma receptor deficient (IFNγR−/−) allogeneic donor T cells result in significantly less graft-versus-host disease (GvHD) than wild-type (WT) T cells, while maintaining an anti-leukemia or graft-versus-leukemia (GvL) effect after allogeneic hematopoietic stem cell transplantation (allo-HSCT). We demonstrated that IFNγR signaling regulates alloreactive T cell trafficking to GvHD target organs through expression of the chemokine receptor CXCR3 in alloreactive T cells. Since IFNγR signaling is mediated via JAK1/JAK2, we tested the effect of JAK1/JAK2 inhibition on GvHD. While we demonstrated that pharmacologic blockade of JAK1/JAK2 in WT T cells using the JAK1/JAK2 inhibitor, INCB018424 (Ruxolitinib), resulted in a similar effect to IFNγR−/− T cells both *in vitro* (reduction of CXCR3 expression in T cells) and *in vivo* (mitigation of GvHD after allo-HSCT), it remains to be determined if *in vivo* administration of INCB018424 will result in preservation of GvL while reducing GvHD. Here, we report that INCB018424 reduces GvHD and preserves the beneficial GvL effect in two different murine MHC-mismatched allo-HSCT models and using two different murine leukemia models (lymphoid leukemia and myeloid leukemia). In addition, prolonged administration of INCB018424 further improves survival after allo-HSCT and is superior to other JAK1/JAK2 inhibitors, such as TG101348 or AZD1480. These data suggest that pharmacologic inhibition of JAK1/JAK2 might be a promising therapeutic approach to achieve the beneficial anti-leukemia effect and overcome HLA-barriers in allo-HSCT. It might also be exploited in other diseases besides GvHD, such as organ transplant rejection, chronic inflammatory diseases and autoimmune diseases.

## Introduction

Allo-HSCT is often the most effective and curative treatment for patients with hematologic malignancies such as relapsed or refractory leukemia and marrow failure states such as myelodysplastic syndromes (MDS), myelofibrosis, and aplastic anemia [1]. The therapeutic benefits of allo-HSCT are primarily derived from an anti-leukemia effect (graft-versus-leukemia effect or GvL) that is mediated by mature donor T cells present in the donor graft. Unfortunately, these donor T cells also induce GvHD, the major life-threatening complication of allo-HSCT [2]. Thus, the clinical goal is to minimize GvHD without abrogating the beneficial GvL effect.

The current treatment of GvHD involves the reduction of T cell numbers or function resulting in loss of donor engraftment, alloreactivity (GvHD), and an anti-leukemia effect (GvL), thereby potentially undermining of the beneficial effects of allo-HSCT leading to enhanced leukemia relapse. Managing the threat of GvHD while maximizing the beneficial GvL effect would broaden the scope and usefulness of allo-HSCT procedures and mitigate the major cause of morbidity and mortality in patients with hematologic malignancies undergoing allo-HSCT. The development of simple and innovative pharmacologic approaches to modulate alloreactive donor T cell trafficking to GvHD target organs without affecting T cell trafficking to leukemia cells *in vivo* represents a significant advance in allo-HSCT prophylaxis.

Recently, we reported that IFNγR is upregulated in activated T cells and that IFNγR−/− allogeneic donor T cells result in significantly less GvHD than WT T cells, while preserving GvL with normal allo-reactivity. In addition, we demonstrated that IFNγR signaling is essential for a chemokine receptor, CXCR3, expression and T cell trafficking to GvHD target organs [3]. IFNγR signaling is mediated via JAK1 and JAK2. INCB018424 is a commercially available potent JAK1/JAK2-specific inhibitor [4], [5], that is FDA-approved for advanced myelofibrosis and currently being tested in clinical trials for the treatment of other myeloproliferative disorders [4], [5]. We found that this small molecule inhibitor dramatically reduces the expression of CXCR3 in both human and murine activated T cells [3]. Most importantly, INCB018424 significantly reduced GvHD and improved survival after allo-HSCT by modulating allogeneic donor T cell trafficking to GvHD target organs as seen in IFNγR−/− T cells [3]. Based on these data, we hypothesize that INCB018424 will preserve the beneficial GvL effect while mitigating GvHD after allo-HSCT.

## Materials and Methods

### Ethics statement

This study was carried out in strict accordance with current National Institutes of Health guidelines. The animal care, use, and euthanasia protocols were approved by the Washington University School of Medicine Animal Studies Committee (Approval Number: 20120058).

### Mice

All mice were obtained from Jackson Laboratory (Bar Harbor, ME).

### Cell culture

Mouse pan T cells (CD4+ and CD8+ T cells) were isolated from mouse spleens using Miltenyi pan T cell isolation kit and an AutoMACS (Miltenyi Biotech, Auburn, CA) [6]. The isolated pan T cells were activated for three days in the presence of anti-CD3/CD28 antibody-coated beads (bead:cell = 1∶1) (Invitrogen, Carlsbad, CA) in Xcyte media with IL-2 (10 IU/ml) [7].

### Flow cytometric analysis

The antibodies used for flow cytometric analyses are as follows. CD4, CD8, H-2K^b^, CD3, B220, CD45.2, STAT1 (clone: 1/Stat1), STAT1 (pY701) (clone: 4a) (BD Pharmingen) and CD183 (clone: CXCR3-173) (eBioscience). All data were collected on a FACScan cytometer (BD Biosciences, Mountain View, CA) and analyzed using FlowJo (Tree Star Inc, Ashland, OR).

### Allo-HSCT

Allo-HSCT was performed as previously described [6], [7]. In brief, 5×10^6^ T cell-depleted bone marrow cells (TCD BM) (CD45.1+ B6 (H-2^b^)) and 5×10^5^ pan T cells (CD45.2+ B6 (H-2^b^)) were injected into lethally irradiated (900–925cGy) Balb/c recipient mice (H-2^d^, CD45.2+). The Balb/c-derived B cell lymphoma cell line, A20, was used in both systemic leukemia and solid tumor models. The primary acute promyelocytic leukemia (APL) cells, which were harvested from the human PML-RARα knock-in mice [8], were used in myeloid leukemia model.

### *In vivo* bioluminescence imaging (BLI)

GvL effect assessment and BLI of animals were done as previously described [6], [9]. BLI was performed once every week starting on day 1 until day 43 post allo-HSCT. Mice were injected intraperitoneally with 150 mg/g D-luciferin (Biosynth, Naperville, IL) in PBS, anesthetized with 2.5% isoflurane, and imaged with a charge-coupled device (CCD) camera-based BLI system (IVIS 100; Caliper, Hopkinton, MA; exposure time 1–60 seconds, binning 16, field of view 12, f/stop 1, open filter. Signal was displayed as photons/sec/cm^2^/sr [9].

### JAK1/JAK2 inhibitors

INCB018424, TG101348, and AZD1480 were purchased from Selleck Chemicals (Houston, TX) and prepared for injection as described in the manufacturer’s instructions.

### Statistical analysis

The significance of differences in survival between treatment groups was analyzed using the log-rank test. For all other analyses, the unpaired *t*-test was used.

## Results

### *In vivo* administration of INCB018424 preserves a strong anti-leukemia effect

**Figure 1:**
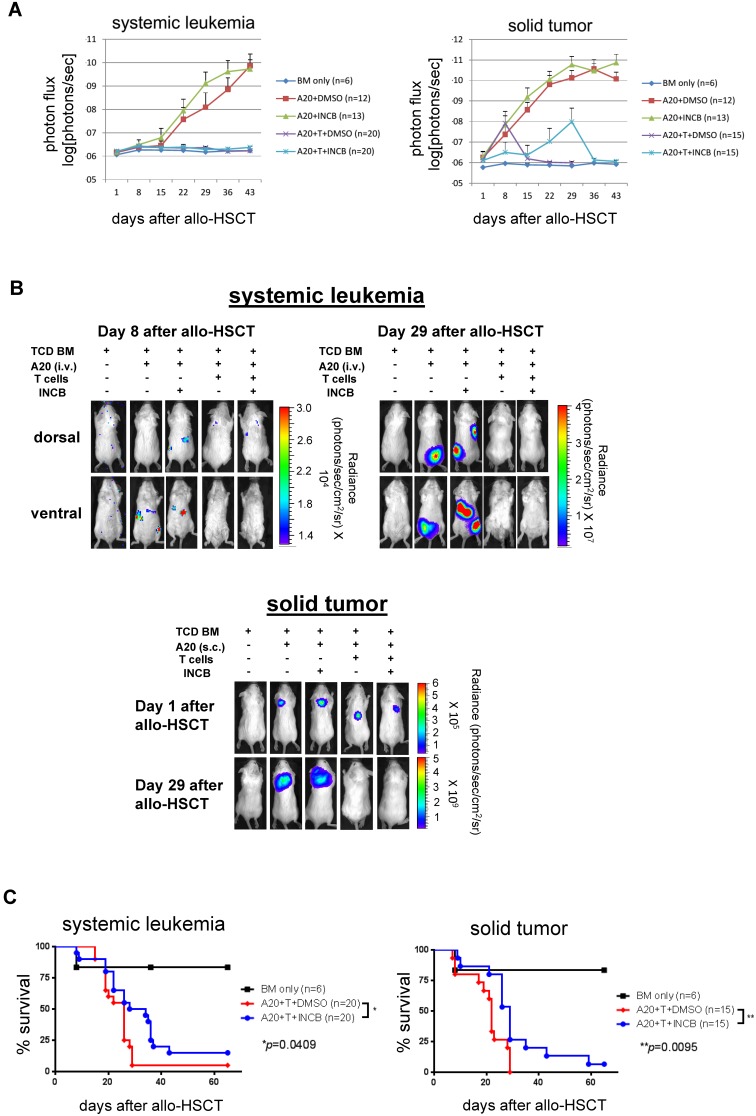
*In* ** *vivo* administration of INCB018424 maintains a beneficial GvL effect and improves survival after allo-HSCT. Allo-HSCT was performed as described in the Materials and Method. CBRluc-expressing A20 B cell lymphoma cells were injected either i.v. (1×10^4^ cells) or s.c. (1×10^5^ cells in 100 ul PBS). *In vivo* BLI imaging technique was used to measure tumor burden once every week starting day 1 to day 43 after allo-HSCT. INCB018424 was injected for three weeks (days 3–23) twice a day (100 µg/injection, i.p.). (**A**) Systemic leukemia (left panel) and solid tumor (right panel) models. Photon flux was measured with a region of interest drawn over the entire body of each mouse. (**B**) Actual images of 1 representative mouse from each group are shown. Data represents the pool of three independent experiments. Systemic A20 lymphoid leukemia model is shown in upper panels and solid tumor A20 lymphoid leukemia model shown in bottom panel. (**C**) Survival of mice shown in Kaplan-Meier plots of both a systemic (left panel) and a solid tumor model (right panel).

Since the clinical goal of allo-HSCT is to prevent GvHD without abrogating GvL, we examined if the *in vivo* administration of INCB018424 preserves the beneficial anti-leukemia effect by performing BLI in two different mouse models of tumor growth; one a systemic leukemia model (**Fig. 1**) and the other a solid tumor lymphoma model (**Fig. 1**) [3], [7]. Using murine T-cell depleted allo-HSCT model, B6 (H-2^b^) → Balb/c (H-2^d^) (925 cGy TBI), with infusion of pan T cells (5×10^5^) (i.v.) and CBRluc-expressing A20 leukemia cells (Balb/c-derived) either i.v. (systemic leukemia) or s.c. (solid tumor) at day 0, followed by administration of INCB018424, we examined the effect of this small molecule inhibitor on GvL. BLI was performed once every week starting day 1 until day 43 (**Fig. 1A, B**).

We found that INCB018424 treatment preserves the potent anti-leukemia effect in both tumor models (**Fig. 1A, B**). These data suggest that pharmacologic modulation of JAK1/JAK2 using INCB018424 would be a promising therapeutic approach to overcome HLA-barriers in allo-HSCT.

### Prolonged administration of INCB018424 prevents GvHD and is superior to other JAK inhibitors

**Figure 2:**
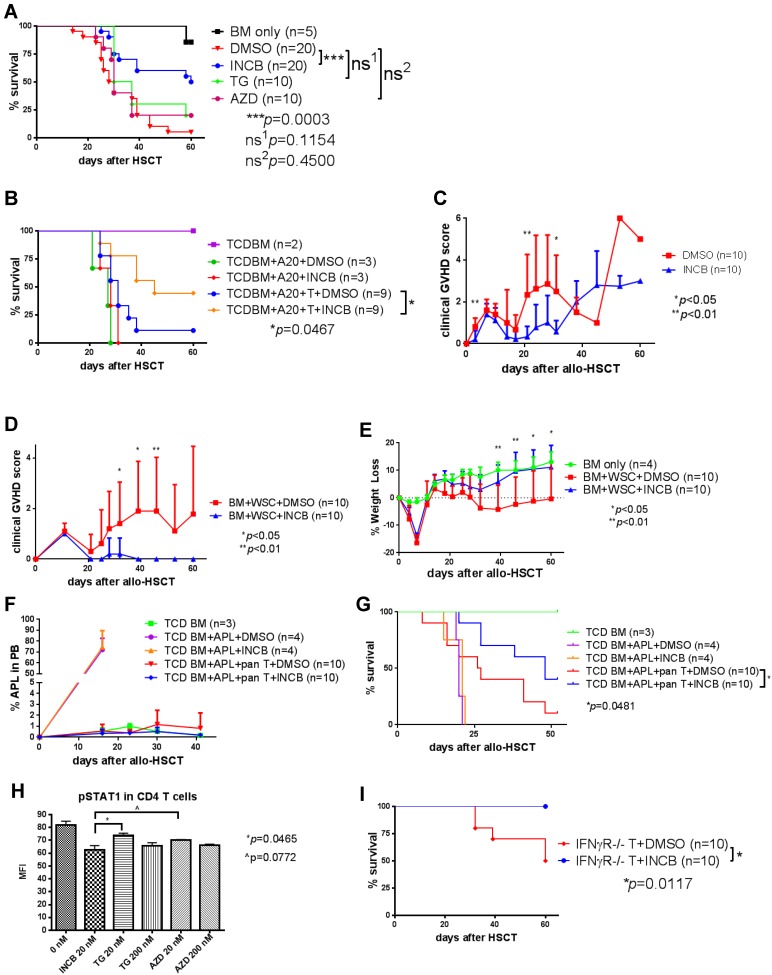
Prolonged administration of INCB018424 further improves survival after allo-HSCT. (**A**) Administration of INCB018424 for one month (days +1–+31) twice a day (100 µg/injection, s.c.) significantly improves survival after allo-HSCT (*p* = 0.0003). (**B–C**) Prolonged administration of INCB018424 improves survival while preserving GvL (**B**). 1×10^5^ A20 cells (i.v) were transplanted along with allogeneic TCD BM and pan T cells at day 0. Clinical GvHD scores according to Cooke et al [10] (**C**). (**D–E**) B6 mice were lethally irradiated (1,200 cGy) at day -1 and injected (i.p.) with 50 µg of anti-NK1.1 mAb (PK136). BM cells (5×10^6^) and whole splenocytes (20×10^6^) harvested from Balb/c were transplanted into lethally irradiated B6 at day 0, followed by INCB018424 for 31 days (days +1–+31). Clinical GvHD scores according to Cooke et al [10] (**D**) and weight change (**E**). (**F–G**) B6 mice were lethally irradiated (1,200 cGy) at day -1 and injected (i.p.) with 50 µg of anti-NK1.1 mAb (PK136). TCD BM (5×10^6^) and pan T cells (5×10^6^) isolated from Balb/c were transplanted into the lethally irradiated B6 at day 0. Banked primary APL cells (5×10^5^) were injected (i.v.) along with TCD BM and pan T cells at day 0. INCB018424 was administered s.c. twice a day for 31 days (days +1–+31). (**F**) Percentage of APL cells in the PB was measured by flow cytometry after staining PB cells starting at day +16 once every week until day +41. Anti-H-2Kb and anti-CD45.2 antibodies were used to identify the APL cells. H-2^b^+ CD45.1+ B6 recipients and H-2^d^+ CD45.2+ Balb/c donors were used to discriminate APL from all other cells. (**G**) Shown is a Kaplan-Meier survival curve. (**H**) INCB018424 is superior to other JAK inhibitors, such as TG101348 and AZD1480, in blocking IFNγR signaling. As an indicator of IFNγR signaling, we assessed STAT1 phosphorylation by intracellular pSTAT1 staining and FACS. MFI: geometric mean fluorescence intensity. (**I**) Lethally irradiated (900 cGy) Balb/c mice were transplanted with TCD BM (5×10^6^) obtained from B6 WT mice and pan T cells (5×10^5^) isolated from B6 WT or IFNγR−/− mice at day 0. INCB018424 was administered s.c. twice a day for 31 days (days +1–+31). INCB018424 treatment for 31 days results in free of GvHD in 90% of recipients and 100% survival when mice are transplanted with IFNγR−/− T cells.

As we previously reported, INCB018424 treatment improves survival by mitigating GvHD after allo-HSCT. Although the survival after INCB018424 treatment was statistically significant in that study, the overall survival rate after INCB018424 (40% at day 35 post allo-HSCT) was not as effective as that seen in IFNγR−/− T cells (80% at day 35 post allo-HSCT) [3]. Likewise, in the current study, we observed a statistically significant improvement of survival after allo-HSCT along with infusion of leukemia cells. However, the overall survivals at day 30 in the systemic leukemia and solid tumor models were 50% (5% survival in the control group) and 26.7% (0% survival in the control group), respectively (**Fig. 1C**). The critical difference between the previous genetic study and the current pharmacologic approach is the duration of JAK1/JAK2 activation. While IFNγR-mediated JAK1/JAK2 signaling in IFNγR−/− T cells is presumably inactive (although JAK1 and JAK2 activation mediated by other signaling pathways might not be affected), the pharmacologic blockade of JAK1/JAK2 in WT T cells by INCB018424 was maintained for only three weeks after allo-HSCT. Thus, we hypothesized that a prolonged administration of INCB018424 (greater than three weeks *in vivo*) would result in improvement in overall survival. As demonstrated in **Fig. 2A**, we found that the prolonged administration of INCB018424 (for one month) significantly improved overall survival (75% at day 30 and 50% at day 60) in this fully MHC-mismatched mouse allo-HSCT model. In addition, this improved survival was similarly observed in A20 bearing mice when these mice were treated for one month with INCB018424 (**Fig. 2B–C**). Furthermore, we observed a similar immunomodulatory effect of INCB018242 in a different mouse model (Balb/c to B6) in terms of overall survival (80% - DMSO vs. 100% - INCB), clinical GvHD score (**Fig. 2D**), and weight change (**Fig. 2E**). Westervelt et al generated an APL mouse model (B6; H-2^b^, CD45.2+) in which the human PML-RARα gene is inserted into the 5′ UTR of the murine cathepsin G gene [8]. These mice develop a fatal myeloid leukemia with 90–100% penetrance [8]. The leukemia is characterized by marked peripheral leukocytosis [8]. Banked primary APL cells (5×10^5^ cells) obtained from these mice were injected (i.v.) into the lethally irradiated B6 recipient mice along with TCD BM (5×10^6^ cells) and splenic pan T cells (5×10^6^ cells) isolated from Balb/c donor mice at day 0. Starting at day 16 post transplantation, we determined leukemic burden of these mice by measuring percent APL cells in the PB once every week. We found that administration of INCB018424 preserves GvL activity in this myeloid leukemia model (**Fig. 2F**) while significantly reducing GvHD (**Fig. 2G**). These data and our previous report [3], in which altered T cell trafficking was found in another allo-HSCT model (FVB to Balb/c) after INCB018424 treatment, suggest that the effect of *in vivo* blockade of JAK1/JAK2 on GvHD and GvL might not be model-specific. We also found that INCB018424 is superior to other JAK inhibitors, such as TG101348 and AZD1480 (**Fig. 2A**). Since IFNγR-mediated activation of JAK1 and JAK2 results in phosphorylation of STAT1, we examined if there are any differences in the ability of these three JAK inhibitors to inhibit STAT1 phosphorylation. We observed that INCB018424 is the most effective at blocking STAT1 phosphorylation (**Fig. 2H**). These data suggest that INCB018424 is superior in inhibiting IFNγR-mediated JAK1/JAK2 signaling and phosphorylation of STAT1 *in vitro* and *in vivo* than the other JAK1/JAK2 inhibitors tested and may potentially be an ideal drug to test in the context of GvHD prophylaxis in human allo-HSCT in the future.

Although we demonstrated that IFNγR signaling can be modulated by targeting JAK1/JAK2, we cannot exclude the possibility that JAK1/JAK2 inhibitors have off-target effects, considering JAK1 and JAK2 function as signal transducers of multiple cytokine receptors and activation pathways. Thus, we examined if INCB018424 could further reduce GVHD in the recipients of IFNγR−/− T cells. Interestingly, INCB018424 administration resulted in 100% survival (**Fig. 2I**) and negligible evidence of clinical GvHD in 90% of the recipients; 1/10 mouse had grade 2 denuded skin based on the Cooke et al. GvHD scoring system [10]. These data suggest that INCB018424 may modulate signaling pathways other than, and in addition to, JAK1/JAK2. Alternatively, JAK1/JAK2 inhibitors perhaps reduce IFNγR signaling in recipients or donor BM-derived antigen presenting cells (APCs), which may also contribute to reduction of MHC I and II expression in recipient APCs [11], [12].

## Discussion

In our previous report, we demonstrated that either genetic deficiency of IFNγR signaling or pharmacologic inhibition of its downstream targets JAK1 and JAK2 using INCB018424 results in less GvHD after allo-HSCT by reducing alloreactive T cell trafficking to GvHD target organs [3]. In the current study, we further demonstrate and confirm that the pharmacologic inhibition of JAK1/JAK2 preserves the beneficial anti-leukemia effect previously reported by our group in IFNγR−/− T cells [3] in two different leukemia models (A20 lymphoid leukemia and APL myeloid leukemia).

We also show that extending the duration of INCB018424 administration by 10 days (from 21 days in our previous study to 31 days in our current study) [3] significantly reduces GvHD and improves the overall survival of major MHC-mismatched allo-HSCT recipients. Spoerl et al. recently reported similar results [13]. While the overall survivals are comparable between the two studies (relative survival rates comparing to the BM only groups: 65% - Spoerl et al. vs 58.3% - current study), there are several differences in the experimental designs of our study and that of Spoerl et al. First, Spoerl et al used a dose of INCB018424 three times higher than that used in our current study (Spoerl et al.: 30 mg/kg/day; current study: equivalent to 10 mg/kg/day with an average body weight of recipient mice, approximately 20 g/mouse). It is possible that this high dose of INCB018424 resulted in the reduction of T cell expansion in their BLI study [13] while our previous study demonstrated that the lower dose of INCB018424 altered T cell trafficking but did not affect T cell expansion [3]. Second, Spoerl et al. used only 60% of the T cell dose used in our study (0.3×10^6^ T cells vs. 0.5×10^6^ T cells). It is possible that this modest difference may also contribute to the observed marginal improved survival reported by Spoerl et al. Third, the duration of INCB018424 administration was from day -1 to day +20 by oral gavage in the Spoerl study, while in our study it was started on day +1 and continued to day +31 by s.c. injection.

There are several possible reasons why INCB018424 might be more effective at reducing GvHD than TG101348 and AZD1480 in spite of them all being potent JAK inhibitors. Since others have shown that plasma concentrations of both TG101348 and AZD1480 are well maintained when administered twice a day and these drugs are effective in reducing tumor burden *in vivo* [14]–[16], we speculate that the drug efficacy and stability *in vivo* is likely not the issue. First, it is conceivable that the preferential inhibition of JAK2 over JAK1 by TG101348 (35 fold) and AZD1480 (5 fold), compared to 1.18 fold with INCB018424, may possibly lead to reduced inhibition of IFNγR signaling (via STAT1) [16]–[18]. Therefore TG101348 and AZD1480 may preferentially inhibit other signaling pathways involving only JAK2, such as IL-12 (via STAT4), leptin (via STAT3), and IL-3, IL-5 or GM-CSF (via STAT5) [19]. Indeed, Dey *et al* demonstrated that IL-12 is protective against GvHD by inhibiting activation of alloreactive CD4+ T cells [20]. In addition, it has been reported that STAT3 signaling is essential for myeloid-derived suppressor cells [21], which may have an important role in the suppressing the proliferation of alloreactive T cells [22]. Furthermore, the Blazar group reported that constitutive activation of STAT5b reduces GvHD [23], suggesting that inhibition of STAT5 by these compounds could aggravate GvHD. Second, it has been shown that AZD1480 and TG101348 can reduce the expression of PD-L1 and PD-L2 [24], [25], which are ligands of programed cell death 1 (PD1). Thus, it is possible that reduction of PD-L1 and PD-L2 on target tissues by AZD1480 might lead to an increase of alloreactive T cells in GvHD target organs [26], [27], offsetting the reduced T cell trafficking to the GvHD target organs. Lastly, there might be off-target effects by these drugs which may be proinflammatory rather than anti-inflammatory.

It has been recently reported that aberrant function of JAK kinases is found often in AML [28], [29], suggesting that JAK1/JAK2 inhibitors may also induce direct negative regulatory effects on AML survival and proliferation. If successful in humans, this strategy (the use of JAK1/JAK2 inhibitors as GvHD prophylaxis for allo-HSCT) may limit the need for expensive and labor intensive *ex vivo* cellular manipulations, such as T cell depletion to reduce GvHD while maintaining GvL. In addition, this approach may also result in the reduced use of post-transplant immunosuppressive agents which, may themselves, contribute to both morbidity and mortality after allo-HSCT. Consistent with our study, Spoerl et al. recently demonstrated that INCB018424 was able to reduce established GVHD of 6/6 patients treated [13], suggesting that blockade of JAK1/JAK2 would be an effective therapeutic strategy for established GvHD as well as a rational approach as GvHD prophylaxis. Furthermore, pharmacologic JAK1/JAK2 blockade, either alone or in conjunction with other methods of GvHD prophylaxis may permit for those patients who lack a human leukocyte antigen (HLA)-matched sibling or matched unrelated donor (approximately 50% of patients) to proceed to transplant with limited GvHD and a well preserved GvL effect. Taken together, our data suggests that JAK1/JAK2 is a promising therapeutic target to prevent GvHD while maintaining GvL.
